# The spatio-temporal relationship between concurrent lesion and brain atrophy changes in early multiple sclerosis: A post-hoc analysis of the REFLEXION study

**DOI:** 10.1016/j.nicl.2023.103397

**Published:** 2023-04-05

**Authors:** Giordano Gentile, Rozemarijn M. Mattiesing, Iman Brouwer, Ronald A. van Schijndel, Bernard M.J. Uitdehaag, Jos W.R. Twisk, Ludwig Kappos, Mark S. Freedman, Giancarlo Comi, Dominic Jack, Frederik Barkhof, Nicola De Stefano, Hugo Vrenken, Marco Battaglini

**Affiliations:** a Department of Medicine, Surgery and Neuroscience, University of Siena, 53100 Siena, Italy; b Siena Imaging SRL, 53100 Siena, Italy; c MS Center Amsterdam, Radiology and Nuclear Medicine, Amsterdam Neuroscience, Amsterdam UMC location VUmc, De Boelelaan 1118, 1081 HZ Amsterdam, the Netherlands; d MS Center Amsterdam, Neurology, Amsterdam Neuroscience, Amsterdam UMC location VUmc, De Boelelaan 1118, 1081 HZ Amsterdam, the Netherlands; e Epidemiology and Data Science, Amsterdam UMC location VUmc, De Boelelaan 1118, 1081 HZ Amsterdam, the Netherlands; f Research Center for Clinical Neuroimmunology, and Neuroscience Basel (RC2NB), University Hospital Basel, CH-4031 Basel, Switzerland; g Neurology Departments of Head, Spine and Neuromedicine, Biomedical Engineering and Clinical Research, University of Basel, Basel, Switzerland; h Department of Medicine, University of Ottawa, Ottawa ON, K1N 6N5, Ontario, Canada; i Ottawa Hospital Research Institute, Ottawa ON, K1H 8L6, Ontario, Canada; j Università Vita Salute San Raffaele, Casa di Cura del Policlinico, 20132 Milan, Italy; k Merck Serono Ltd, Feltham, TW14 8HD, UK, an affiliate of Merck KGaA; l UCL Institutes of Neurology and Healthcare Engineering, London, WC1E 6BT, UK

**Keywords:** Brain atrophy, Interferon β-1a, Magnetic resonance imaging, Early multiple sclerosis, White matter lesions

## Abstract

**Background:**

White matter (WM) lesions and brain atrophy are present early in multiple sclerosis (MS). However, their spatio-temporal relationship remains unclear.

**Methods:**

Yearly magnetic resonance images were analysed in 387 patients with a first clinical demyelinating event (FCDE) from the 5-year REFLEXION study. Patients received early (from baseline; N = 258; ET) or delayed treatment (from month-24; N = 129; DT) with subcutaneous interferon beta-1a. FSL-SIENA/VIENA were used to provide yearly percentage volume change of brain (PBVC) and ventricles (PVVC). Yearly total lesion volume change (TLVC) was determined by a semi-automated method. Using linear mixed models and voxel-wise analyses, we firstly investigated the overall relationship between TLVC and PBVC and between TLVC and PVVC in the same follow-up period. Analyses were then separately performed for: the untreated period of DT patients (first two years), the first year of treatment (year 1 for ET and year 3 for DT), and a period where patients had received at least 1 year of treatment (stable treatment; ET: years 2, 3, 4, and 5; DT: years 4 and 5).

**Results:**

Whole brain: across the whole study period, lower TLVC was related to faster atrophy (PBVC: B = 0.046, SE = 0.013, p < 0.001; PVVC: B = −0.466, SE = 0.118, p < 0.001). Within the untreated period of DT patients, lower TLVC was related to faster atrophy (PBVC: B = 0.072, SE = 0.029, p = 0.013; PVVC: B = −0.917, SE = 0.306, p = 0.003). A similar relationship was found within the first year of treatment of ET patients (PBVC: B = 0.081, SE = 0.027, p = 0.003; PVVC: B = −1.08, SE = 0.284, p < 0.001), consistent with resolving oedema and pseudo-atrophy.

Voxel-wise: overall, higher TLVC was related to faster ventricular enlargement. Lower TLVC was related to faster widespread atrophy in year 1 in both ET (first year of treatment) and DT (untreated) patients. In the second untreated year of DT patients and within the stable treatment period of ET patients (year 4), faster periventricular and occipital lobe atrophy was associated with higher TLVC.

**Conclusions:**

WM lesion changes and atrophy occurred simultaneously in early MS. Spatio-temporal correspondence of these two processes involved mostly the periventricular area. Within the first year of the study, in both treatment groups, faster atrophy was linked to lower lesion volume changes, consistent with higher shrinking and disappearing lesion activity. This might reflect the pseudo-atrophy phenomenon that is probably related to the therapy driven (only in ET patients, as they received treatment from baseline) and “natural” (both ET and DT patients entered the study after a FCDE) resolution of oedema. In an untreated period and later on during stable treatment, (real) atrophy was related to higher lesion volume changes, consistent with increased new and enlarging lesion activity.

## Highlights

• White matter lesion changes and brain atrophy occurred simultaneously in early MS.
• Spatio-temporal correspondence involved mostly the periventricular region.
• In year 1, lower lesion volume changes were related to faster pseudo-atrophy.
• Pseudo-atrophy was linked to the therapy-driven and “natural” resolution of oedema.
• In the untreated period, higher lesion volume changes were linked to faster atrophy.

## 1 Introduction

Multiple sclerosis (MS) is a chronic demyelinating disease of the central nervous system characterised by the concurrent presence of focal areas of inflammation in the white (WM) and grey (GM) matter (lesions), and diffuse damage and neurodegeneration across the whole brain (atrophy) (Lassmann et al., 2007). Although these two processes are present early in the disease course of MS, the dynamics of accumulation of WM lesions and brain atrophy are not completely understood.

To date, the few longitudinal magnetic resonance imaging (MRI) studies investigating the relationship between WM lesion activity and brain atrophy have focused on the assumption that inflammation precedes neurodegeneration (Chard et al., 2003, Dalton et al., 2002, Paolillo et al., 2004). However, these two biological processes could represent somewhat unrelated aspects of the disease (Tauhid et al., 2014) working in parallel, with one prevailing over the other at different stages of disease or in different brain regions (Bodini et al., 2009, Bodini et al., 2016). Indeed, weak-to-modest associations between the development of regional brain atrophy and lesion changes in number (newly detected lesions) and volume (progressive tissue damage in pre-existing lesions) suggest that lesion activity contributes only partially to brain atrophy (Battaglini et al., 2009, Cappellani et al., 2014, Roosendaal et al., 2011). At present, only a few studies have investigated how WM lesion changes (activity) and brain volume changes (atrophy) are linked within the same time interval (e.g., Richert et al., 2006, Sailer et al., 2001). Furthermore, studies within early MS are even more scant (Dalton et al., 2004, Varosanec et al., 2015). Finally, the studies employing voxel-wise analyses in early MS focused, to the best of our knowledge, on the longitudinal spatial correlation between WM and GM damage (Raz et al., 2010, Rocca et al., 2016) but did not examine the relationship between WM lesion activity and whole brain atrophy.

To develop more targeted therapeutic strategies which can effectively intervene in the early stage of disease, it is crucial to better understand the underlying disease mechanisms, how these relate to disease progression, and whether these can be either modified by treatment or disease worsening. Thus, it is of great relevance to investigate whether inflammation and brain atrophy are two independent processes which might develop simultaneously within early MS. Furthermore, exploring the concurrent spatio-temporal evolution of these two pathological processes will provide additional information that could have important implications for the design of clinical trials and for the interpretation of data in both clinical and research settings.

Longitudinal MRI studies are mandatory to explore the dynamic associations between WM lesions and brain atrophy. The randomised, double-blind, placebo-controlled, multicentre REFLEX clinical trial and its extension, REFLEXION (REbif FLEXible dosing in early MS extensION), provided such an opportunity. In this trial, patients presenting with a first clinical demyelinating event (FDCE) were followed over a period of five years with yearly MRI scans. Primary analyses on the REFLEXION study showed how treatment with subcutaneous interferon beta-1a (sc IFN β-1a) was associated with reduced MRI activity (Comi et al., 2012, Comi et al., 2017). However, the link between atrophy and WM lesions was not investigated.

In this study we investigated whether, in early MS, WM lesion activity and brain atrophy were spatially interconnected within the same follow-up period and analysed whether these two processes developed simultaneously over time. In addition, we investigated how treatment influenced the concurrent relationship between WM lesion activity and brain atrophy. To this respect, we first analysed whether WM lesion activity and brain atrophy were differentially related prior to and after treatment onset. Secondly, we examined how these two processes were associated within the first year of treatment. Thirdly, we explored how WM lesion activity and brain atrophy were linked during a stable treatment period. Finally, we assessed whether the relationship between WM lesion activity and brain atrophy differed between patients who converted to MS and those who did not.

## 2 Methods

### 2.1 Population

REFLEXION was a pre-planned extension of the Phase III trial REFLEX, the procedures and design of which have been described in detail elsewhere (Comi et al., 2012, Comi et al., 2017). Briefly, patients were eligible if they had experienced a FCDE suggestive of MS within 60 days prior to study entry and if they met the criteria of having at least two clinically silent lesions of 3 mm or more on a T2-weighted brain MRI scan, at least one of which was ovoid, periventricular, or infratentorial. At the start of the REFLEX trial, patients were randomised to three treatment arms; early treatment (ET) with either sc IFN β-1a 44 µg once a week or three times a week, or delayed treatment (DT), where patients received placebo. At the start of REFLEXION, patients already receiving treatment with sc IFN β-1a continued with the same treatment, and patients in the DT group were switched to treatment with sc IFN β-1a 44 µg three times a week. In this study, the two sc IFN β-1a dosing arms (once a week and three times a week) were merged. Clinically definite MS (CDMS) was defined as a relapse accompanied by an abnormal MRI scan, or a sustained increase in Expanded Disability Status Scale score of ≥ 1.5 points. For each patient, the yearly interval-specific CDMS status was provided. If patients converted to CDMS they switched to open-label treatment with sc IFN β-1a three times a week.

### 2.2 Ethics approval

This post-hoc study used data from the REFLEX and REFLEXION trials, which were undertaken in compliance with the Declaration of Helsinki and standards of Good Clinical Practice according to the International Conference on Harmonisation of Technical Requirements for Registration of Pharmaceuticals for Human Use. Before initiation of the trials at each study site, the relevant institutional review board or independent ethics committee reviewed and approved the trial protocols, patient information leaflets, informed consent forms, and investigator brochures. All patients provided written informed consent at the screening visit of REFLEX, and before enrolment to REFLEXION.

### 2.3 MRI data

In this post-hoc analysis of the 5-year REFLEXION study, multicentre yearly MRI scans consisting of 2D dual-echo 1x1x3 mm^3^ proton-density (PD)-/T2- and T1-weighted images, were provided by Calyx/formerly PAREXEL International Corporation. All study sites were required to follow an MRI acquisition protocol to ensure standardised scanning and image acquisition. The protocol specified a preference for 1.5 Tesla scanners, with either GE, Siemens, and Philips scanners being used (Comi et al., 2012). Full scanning details are listed in Supplementary Material Table 1. Manual delineations of PD-/T2-weighted lesions for each yearly visit, and manually edited brain extraction masks were provided by the Image Analysis Center (Amsterdam UMC location VUmc, Amsterdam, the Netherlands). These manual extractions were originally obtained using the Functional MRI of the Brain (FMRIB) software library (FSL) (Smith et al., 2004) brain extraction tool (Smith, 2002) on T1-weighted images. All scans of sufficient quality and passing pre-specified quality control measures (specific criteria of which are described in more detail in the Results section 3.1) were included in the analysis.

### 2.4 MRI analysis

#### 2.4.1 Longitudinal atrophy quantification

Corrections for slice-to-slice variations (interleaved acquisition) and subsequent lesion filling with the linearly registered PD-/T2-weighted manual delineations were conducted on the T1-weighted images. Using the resulting T1-weighted images, and manually edited brain masks as input, FSL-SIENA (Smith et al., 2001, Smith et al., 2002) and its extension VIENA (Vrenken et al., 2014), were used to estimate yearly percentage brain volume change (PBVC) and percentage ventricular volume change (PVVC). Longitudinal measures of yearly global atrophy and central atrophy were determined using the PBVC and PVVC outputs, respectively. Briefly, more negative PBVC values reflect faster global atrophy, whereas more positive PVVC values reflect faster ventricular enlargement (i.e., central atrophy).

#### 2.4.2 Longitudinal lesion change quantification

An in-house developed method based on subtraction images (Battaglini et al., 2014, Moraal et al., 2010a, Moraal et al., 2010b) was used to segment yearly lesion changes. Details of this method are described in Battaglini et al. (2022). Using this semi-automated method, the slice-to-slice variations in signal intensity on the PD-weighted images were corrected before creating the subtraction images. The PD images of two visits within a 1-year period (e.g., baseline-month 12) were registered to a common halfway space using procedures similar to those utilised by FSL-SIENA software. The PD-weighted image of the first visit of each interval (e.g., baseline) was subtracted from the second visit (e.g., month 12) in order to obtain the subtraction images. For robustness, the subtraction images were further normalised to account for study site variations and differences in the MRI scanners from which the PD images were obtained, by converting all voxel intensities to Z-scores based on the mean and standard deviation (SD) within the non-lesional brain tissue of the respective subtraction image. For this analysis, we labelled any voxels inside the lesion masks with a normalised intensity difference that exceeded 1.5 standard deviations (|Z| > 1.5) as changing. Using baseline and follow-up lesion masks and voxel-wise lesion changes, all individual lesions were labelled as new, enlarging, shrinking, or disappearing. This labelling was then used to calculate the yearly total lesion volume change (TLVC) by subtracting the sum of negative lesion volume changes (shrinking + disappearing) from the positive lesion volume changes (new + enlarging) for each 1-year period.

### 2.5 Voxel-wise input images

To produce the input images for the voxel-wise analyses, a series of tasks were performed. To begin the process, a study-specific template was created. FSL-SIENAX, a software to estimate total brain tissue volume (Smith et al., 2002), was used to obtain the normalised brain volume for all baseline T1-weighted images. Based on the percentile normalised brain volume distribution (from 1st to the 100th percentile), 100 patients were selected. For each of these 100 patients, the T1-weighted images were intensity normalised (divided by the 99th intensity percentile of the non-zero voxels and multiplied by 10,000) and non-linearly registered to the Montreal Neurological Institute (MNI) standard space (voxel size of 2x2x2 mm^3^) using FMRIB’s nonlinear image registration tool (FNIRT) (Andersson et al., 2010); images were then averaged to create the study-specific template.

In order to avoid interpolation bias and to ensure that all images underwent the same pre-processing procedure, a patient-specific template was created. For each patient, T1-weighted images underwent intensity normalisation using the N4 algorithm (Tustison et al., 2010) before linear registration to the baseline scan using FMRIB’s linear image registration tool (FLIRT) (Jenkinson & Smith, 2001). The sum of these T1-weighted scans was averaged to create the patient-specific template. The patient-specific template was then non-linearly registered on the study-specific template and the warp files generated from this step were used for the registration of SIENA outputs on the study-specific template.

To allow voxel-wise analyses of atrophy across subjects, brain edge displacement maps were created in order to study local atrophy. For each patient, the yearly brain edge displacement images provided by SIENA were spatially dilated, non-linearly registered to the study-specific template using the warp field files previously generated, masked with a standard space brain edge image, and smoothed with an isotropic Gaussian kernel with a sigma of 5 mm before re-masking was conducted (Bartsch et al., 2006, De Stefano et al., 2003).

### 2.6 Study design

Population subgroups were formed to address the following research questions at both the whole brain and voxel-wise levels:

1. Investigate the relationship between lesion activity and brain atrophy within the same year. All available data points were analysed.
2. Analyse whether this relationship was different between treatment groups (all available data points were analysed). In this regard, we further investigated the relationship between lesion activity and brain atrophy during:
  a. An untreated period: years 1 and 2 (REFLEX period) of DT patients were analysed, while excluding the interval-specific converters to CDMS. We also investigated whether this relationship differed during the treated period; for this purpose, years 4 and 5 (REFLEXION period) of the DT patients were analysed.
  b. The first year of treatment: year 1 for ET patients and year 3 for DT patients were analysed. Any DT patients who converted to CDMS within the period of the REFLEX study were excluded from this analysis (as per the study protocol, DT patients who converted to CDMS during the first two years received treatment upon conversion, which in their case was earlier than year 3).
  c. A stable treatment period. To prevent the confounding effects of resolving oedema and pseudo-atrophy at the start of treatment, the data points where patients had received at least one year of treatment were analysed (ET: years 2, 3, 4, and 5; DT: years 4 and 5).
3. Assess whether lesion activity and brain atrophy differed between converters and non-converters to CDMS. All available data points were analysed.

### 2.7 Statistical analyses

*Whole brain:* Statistical analyses for the whole brain measures were conducted in Rstudio using linear mixed models to account for the dependency of the yearly repeated measurements of brain atrophy. A three-level structure, in which observations were clustered within patients, and the patients were clustered within the different study sites was used for the analyses. All linear mixed models were corrected for age and sex and we used an alpha level of 0.05 as the cut-off for significance. Full model equations are listed in Supplementary Material Table 2. To address research question 1, we applied a linear mixed model with PBVC or PVVC as the dependent variable and TLVC as the independent variable, while also inserting treatment and the interval-specific CDMS status as additional fixed factors. For research questions 2 and 3, we used a similar linear mixed model but now we also incorporated an interaction between TLVC and treatment (TLVC*treatment) and between TLVC and interval-specific CDMS status (TLVC*CDMS status), respectively. To address research question 2a, we incorporated an interaction between TLVC and period (TLVC*period) in a separate linear mixed model and the interval-specific CDMS status was inserted as covariate for the treated period of DT patients. The same linear mixed model of research question 2 was used for research questions 2b and 2c.

*Voxel-wise:* Permutation testing (Nichols & Holmes, 2002) (5000 permutations), as implemented in the FSL “randomise” program, was used to determine regional statistical inference for each yearly MRI interval. Design matrices within the GLM framework were used with age, sex, and study site as covariates, and the Threshold-Free Cluster Enhancement “randomise” option was used. The voxel-wise analyses were carried out using the edge displacement maps of the entire brain as input images for the “randomise” tool (i.e., the dependent variable). Within these brain edge displacement maps, the “randomise” tool detects regions where brain edge shifts (i.e., atrophy) correlate with lesion activity. Voxel-wise statistics replicated the whole brain analyses but were performed for each yearly MRI interval. To address research question 1, TLVC was used as a regressor with treatment and the interval-specific CDMS status as additional covariates. For research questions 2 and 3, we used similar voxel-wise statistics but now incorporating an interaction between TLVC and treatment (TLVC*treatment) and between TLVC and interval-specific CDMS status (TLVC*CDMS status), respectively. To address research question 2a, TLVC was used as a regressor with the interval-specific CDMS status being inserted as covariate for the treated period of DT patients. The same model was used for research questions 2b and 2c. Only results with at least 15 significant voxels (p < 0.05) were reported. The anatomical location where brain atrophy significantly correlated with lesion activity was determined by using predefined standard space masks (https://www.fmrib.ox.ac.uk/fsl/) as provided by the MNI structural atlas. The following brain areas were considered: infratentorial, periventricular, frontal, occipital, parietal, and temporal lobes. The number (V), location, and mean regression coefficients (β) of significant voxels were reported.

## 3 Results

### 3.1 Population

**Fig. 1:**
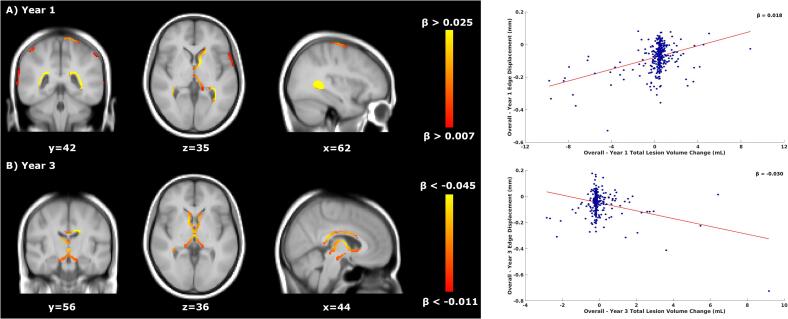
Overall relationship between total lesion volume change and concurrent brain atrophy. Left: voxel-wise analyses within years 1 and 3. Yellow-orange shows the β values (i.e., the regression coefficients) of significant regions where lower TLVC was related to faster (pseudo)atrophy in year 1 (A, top row) and where higher TLVC was related to faster atrophy in year 3 (B, bottom row). Right: the red line shows the mean voxel-wise regression between the TLVC (x-axis) and the mean edge displacement of the significant voxels from the voxel-wise analysis (y-axis). The more negative the edge displacement is, the faster the atrophy. In year 1, for each decrease of 1 mL in TLVC, the edge displacement reduces by 0.018 mm (mean β of the significant voxels). In year 3, for each increase of 1 mL in TLVC, the edge displacement reduces by 0.030 mm. TLVC = total lesion volume change.

**Fig. 2:**
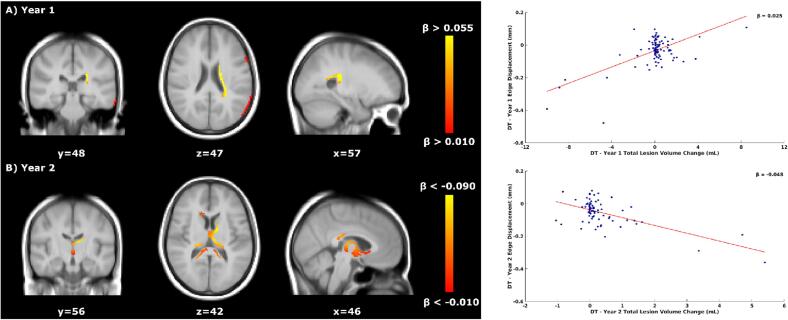
Relationship between total lesion volume change and concurrent brain atrophy in DT patients’ untreated period. Left: voxel-wise analyses within the first two years of the DT patients’ untreated period. Yellow-orange shows the β values (i.e., the regression coefficients) of significant regions where lower TLVC was related to faster (pseudo)atrophy in year 1 (A, top row) and where higher TLVC was related to faster atrophy in year 2 (B, bottom row). Right: the red line shows the mean voxel-wise regression between the TLVC (x-axis) and the mean edge displacement of the significant voxels from the voxel-wise analysis (y-axis). The more negative the edge displacement is, the faster the atrophy. In year 1, for each decrease of 1 mL in TLVC, the edge displacement reduces by 0.025 mm (mean β of the significant voxels). In year 2, for each increase of 1 mL in TLVC, the edge displacement reduces by 0.048 mm. DT = delayed treatment, TLVC = total lesion volume change.

In total, 400 patients were enrolled in the REFLEXION study and provided MRI data during the 5-year trial period. Following the study, data for eight patients were excluded from the analysis: four with incomplete trial data, two with an inconsistent acquisition protocol, and two with no consecutive MRI data. Some visit data were also excluded: five for incorrect/incomplete image(s), eight due to movement in the images, six cases of missing data, and two due to corrupted PD-weighted images. The quality control measures resulted in a total of 158 excluded yearly MRI measurements across the 5-year study period (23 lesion change quantification, 133 longitudinal atrophy, 2 shared rejections). The reasons for failing the quality control measures were low quality images, artefacts, and failure of the pipeline for assessing longitudinal brain changes (e.g., incorrect brain extraction, registration, and tissue segmentation errors). After this quality control step, five subjects with no lesion change or atrophy measures available across the whole study period were excluded. In the end, of the initially 400 patients, 97% (387/400) were included in this analysis. Baseline demographics are shown in Table 1. Yearly atrophy and lesion volume change measures are depicted in Table 2. Longitudinal atrophy and lesion volume change measures across treatment and conversion groups are depicted in the Supplementary Material Figs. 1 and 2.

**Table 1:** Baseline demographics of the included patients.

	Converters to CDMS	Non-converters to CDMS	Early treatment	Delayed treatment	Overall
Patients (N)	160	227	258	129	387
Sex, female, n (%)	94 (58.7)	145 (63.9)	160 (62)	79 (61.2)	239 (61.7)
Age, years (mean ± SD)	30.35 ± 8.03	32.28 ± 8.49	31.7 ± 8.44	31.04 ± 8.17	31.48 ± 8.35
Baseline T2 Lesion Number (mean ± SD)	25 ± 21	21 ± 19	23 ± 20	20 ± 20	22 ± 20
Baseline T2 Lesion Volume (mL) (mean ± SD)	3.88 ± 4.23	3.15 ± 3.68	3.58 ± 3.95	3.19 ± 3.88	3.45 ± 3.93

CDMS = clinically definite multiple sclerosis (across the whole study period), N/n = number, SD = standard deviation.

**Table 2:** Longitudinal atrophy and lesion volume change measures in each year of the study.

Population	Time Interval	PBVC (%/y, mean ± SD)	PVVC (%/y, mean ± SD)	TLVC (mL/y, mean ± SD)
Entire dataset	Year 1	−0.47 ± 0.70	6.01 ± 7.74	−0.22 ± 1.75
	Year 2	−0.41 ± 0.66	2.94 ± 5.16	0.21 ± 0.95
	Year 3	−0.34 ± 0.56	2.88 ± 5.07	0.18 ± 0.93
	Year 4	−0.37 ± 0.57	2.31 ± 4.70	0.18 ± 1.07
	Year 5	−0.40 ± 0.57	2.31 ± 4.20	0.14 ± 0.85
ET and DT patients	Year 1	ET: −0.54 ± 0.71 DT: −0.34 ± 0.67	ET: 6.86 ± 7.18 DT: 4.31 ± 8.53	ET: −0.34 ± 1.6 DT: −0.01 ± 2.01
	Year 2	ET: −0.35 ± 0.60 DT: −0.53 ± 0.75	ET: 2.38 ± 4.58 DT: 4.04 ± 6.00	ET: 0.22 ± 1.00 DT: 0.20 ± 0.83
	Year 3	ET: −0.32 ± 0.54 DT: −0.37 ± 0.59	ET: 2.53 ± 4.22 DT: 3.60 ± 6.39	ET: 0.23 ± 0.87 DT: 0.07 ± 1.02
	Year 4	ET: −0.31 ± 0.58 DT: −0.50 ± -0.53	ET: 2.00 ± 4.74 DT: 2.90 ± 4.43	ET: 0.15 ± 1.05 DT: 0.22 ± 1.11
	Year 5	ET: −0.39 ± 0.57 DT: −0.41 ± 0.57	ET: 2.53 ± 4.30 DT: 1.82 ± 3.94	ET: 0.19 ± 0.89 DT: 0.03 ± 0.71
Conv and Nonc patients	Year 1	Conv: −0.73 ± 0.84 Nonc: −0.43 ± 0.67	Conv: 7.22 ± 7.15 Nonc: 5.84 ± 7.82	Conv: −0.08 ± 1.67 Nonc: −0.25 ± 1.77
	Year 2	Conv: −0.52 ± 0.75 Nonc: −0.37 ± 0.62	Conv: 4.27 ± 6.16 Nonc: 2.44 ± 4.65	Conv: 0.19 ± 0.97 Nonc: 0.22 ± 0.94
	Year 3	Conv: −0.36 ± 0.63 Nonc: −0.32 ± 0.52	Conv: 2.92 ± 6.44 Nonc: 2.87 ± 4.20	Conv: 0.31 ± 1.25 Nonc: 0.10 ± 0.70
	Year 4	Conv: −0.48 ± 0.64 Nonc: −0.31 ± 0.52	Conv: 3.29 ± 5.55 Nonc: 1.73 ± 3.94	Conv: 0.35 ± 1.52 Nonc: 0.07 ± 0.67
	Year 5	Conv: −0.44 ± 0.65 Nonc: −0.37 ± 0.51	Conv: 2.72 ± 5.19 Nonc: 2.06 ± 3.43	Conv: 0.25 ± 1.17 Nonc: 0.07 ± 0.54

DT patients were untreated in years 1 and 2 and treated from year 3 to year 5. Conv = converters to clinically definite multiple sclerosis (CDMS), DT = delayed treatment, ET = early treatment, Nonc = non-converters to CDMS, PBVC = percentage brain volume change, PVVC = percentage ventricular volume change, SD = standard deviation, TLVC = total lesion volume change.

### 3.2 Research question 1: Relationship between atrophy and concurrent lesion activity

All the data points available from the entire dataset of 387 patients were analysed (Table 1).

*Whole brain:* A significant positive relationship between PBVC and TLVC (B = 0.046, SE = 0.013, p < 0.001) was found, consistent with faster global atrophy being related to lower TLVC. A significant negative relationship between PVVC and TLVC (B = −0.466, SE = 0.118, p < 0.001) was found, consistent with faster ventricular enlargement being related to lower TLVC.

*Voxel-wise*: Results are summarised in Table 3. In year 1 (Fig. 1), faster atrophy was associated with lower TLVC (consistent with increased shrinking and disappearing lesion activity). From year 2 to year 5 (Fig. 1), faster ventricular enlargement was related to higher TLVC (consistent with increased new and enlarging lesion activity). Supplementary MaterialFig. 3 shows the p-value maps and the location of the peak values.

**Table 3:** Schematic representation of the voxel-wise significant results for the overall concurrent relationship between atrophy and TLVC.

Time Interval	Number of significant voxels	Location of significant voxels	MNI coordinates (X/Y/Z) of peak location	Mean β value (relationship)
Year 1	4868	PV/FL/PL/TL	62/42/35	0.018 (Faster Atrophy/Lower TLVC)
Year 2	220	PV	48/45/43	−0.027 (Faster Atrophy/Higher TLVC)
Year 3	2629	PV	44/56/36	−0.030 (Faster Atrophy/Higher TLVC)
Year 4	87	OL	70/23/45	−0.021 (Faster Atrophy/Higher TLVC)
Year 5	121	PV	51/78/36	−0.033 (Faster Atrophy/Higher TLVC)

PV = periventricular, FL = frontal lobe, OL = occipital lobe, PL = parietal lobe, TL = temporal lobe, TLVC = total lesion volume change.

### 3.3 Research question 2: Influence of treatment on the relationship between atrophy and concurrent lesion activity

All the data points available from the entire dataset of 387 patients were analysed.

*Whole brain:* ET and DT patients showed significantly different relationships between PVVC and TLVC (p < 0.001). The model only revealed a significant negative relationship for the ET patients (B = −0.781, SE = 0.149, p < 0.001).

*Voxel-wise*: Results are summarised in Table 4. In years 1, 4, and 5, ET and DT patients showed significantly different relationships between atrophy and TLVC. In particular, faster ventricular enlargement, infratentorial, and frontal lobe atrophy was associated with lower TLVC in DT patients. Within the same years and regions, faster atrophy was associated with higher TLVC in ET patients. In year 3, faster ventricular enlargement was related to higher TLVC in both ET and DT patients, but the relationship was stronger for DT. Supplementary MaterialFig. 4 shows the p-value maps and the location of the peak values.

**Table 4:** Schematic representation of the voxel-wise significant results for the influence of treatment on the concurrent relationship between atrophy and TLVC.

Time Interval	Number of significant voxels	Location of significant voxels	MNI coordinates (X/Y/Z) of peak location	ET patients mean β value (relationship)	DT patients mean β value (relationship)
Year 1	77	INF	33/23/9	−0.019 (Faster Atrophy/Higher TLVC)	0.026 (Faster Atrophy/Lower TLVC)
Year 3	446	PV	43/55/37	−0.005 (Faster Atrophy/Higher TLVC)	−0.079 (Faster Atrophy/Higher TLVC)
Year 4	172	PV	52/40/42	−0.011 (Faster Atrophy/Higher TLVC)	0.088 (Faster Atrophy/ Lower TLVC)
Year 5	643	PV/FL	50/43/43	−0.001 (Faster Atrophy/Higher TLVC)	0.082 (Faster Atrophy/ Lower TLVC)

INF = infratentorial, PV = periventricular, FL = frontal lobe, TLVC = total lesion volume change.

### 3.4 Research question 2a: Untreated versus treated period of DT patients

Data points from 97 DT patients (mean age ± SD: 31.65 ± 8.25, number of male/female: 36/61) were analysed within the untreated period. During the treated period, data points from 101 DT patients (mean age ± SD: 31.06 ± 8.17, number of male/female: 41/60) were analysed. In all, 57 patients did not convert to CDMS.

*Whole brain:* During the untreated period (i.e., first two years), DT patients showed a significantly positive relationship between PBVC and TLVC (B = 0.072, SE = 0.029, p = 0.013) and a negative relationship between PVVC and TLVC (B = −0.917, SE = 0.306, p = 0.003). No significant association was reached for the DT patients during the treated period. Overall, similar relationships between WM lesion activity and brain atrophy were observed throughout the untreated and treated periods for DT patients.

**Fig. 3:**
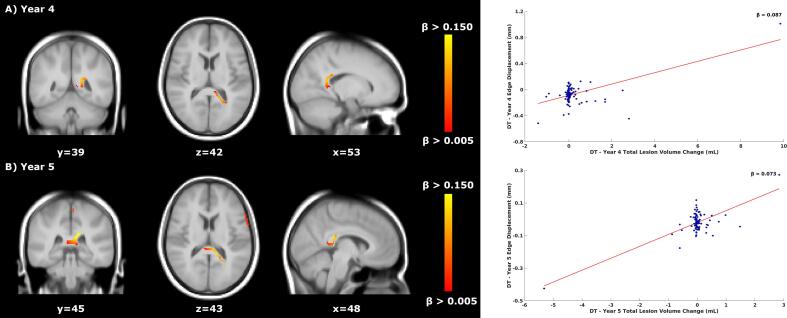
Relationship between total lesion volume change and concurrent brain atrophy in DT patients’ treated period. Left: voxel-wise analyses within the DT patients’ treated period. Yellow-orange shows the β values (i.e., the regression coefficients) of significant regions where lower TLVC was related to faster atrophy in years 4 (A, top row) and 5 (B, bottom row). Right: the red line shows the mean voxel-wise regression between the TLVC (x-axis) and the mean edge displacement of the significant voxels from the voxel-wise analysis (y-axis). The more negative the edge displacement is, the faster the atrophy. For each decrease of 1 mL in TLVC, the edge displacement reduces by 0.087 mm and by 0.073 (mean β of the significant voxels) in years 4 and 5, respectively. DT = delayed treatment, TLVC = total lesion volume change.

*Voxel-wise*: Results are summarised in Table 5. In year 1, faster atrophy was associated with lower TLVC in the untreated period of DT patients. The opposite was found in year 2, where faster ventricular enlargement was related to higher TLVC (Fig. 2). During the treated period of DT patients (Fig. 3), faster ventricular enlargement (years 4 and 5), frontal, and temporal lobe atrophy (year 5) were related to lower TLVC. Supplementary MaterialFig. 5 shows the p-value maps and the location of the peak values.

**Table 5:** Schematic representation of the voxel-wise significant results for the concurrent relationship between atrophy and TLVC during the untreated and treated periods of DT patients.

Time Interval	Number of significant voxels	Location of significant voxels	MNI coordinates (X/Y/Z) of peak location	Mean β value (relationship)
Year 1 (untreated)	2034	PV/PL/TL/INF	57/48/47	0.025 (Faster Atrophy/Lower TLVC)
Year 2 (untreated)	1464	PV	46/56/42	−0.048 (Faster Atrophy/Higher TLVC)
Year 4 (treated)	223	PV	53/39/42	0.087 (Faster Atrophy/Lower TLVC)
Year 5 (treated)	1000	PV/FL/TL	48/45/43	0.073 (Faster Atrophy/Lower TLVC)

INF = infratentorial, PV = periventricular, FL = frontal lobe, PL = parietal lobe, TL = temporal lobe, TLVC = total lesion volume change.

### 3.5 Research question 2b: First year of treatment

Data points from 231 ET patients (mean age ± SD: 31.9 ± 8.38, number of male/female: 82/149) and 65 DT patients (mean age ± SD: 31.72 ± 7.62, number of male/female: 24/41) were analysed. In all, 267 patients did not convert to CDMS (208 ET patients and 59 DT patients).

*Whole brain:* ET and DT patients showed significantly different relationships between PBVC and TLVC in the first year of treatment (p = 0.002). The model revealed that the direction of the relationship was different: positive for ET patients (B = 0.081, SE = 0.027, p = 0.003) and negative for DT patients (B = −0.141, SE = 0.065, p = 0.032). Similar results were found for the relationship between PVVC and TLVC, with ET and DT showing a significant difference in the first year of treatment (p < 0.001). The model revealed that the direction of the relationship was different: negative for ET patients (B = −1.08, SE = 0.284, p < 0.001) and positive for DT patients (B = 3.41, SE = 0.677, p < 0.001).

**Fig. 4:**
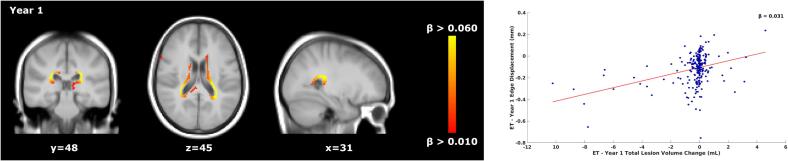
Relationship between total lesion volume change and concurrent brain atrophy in ET patients’ first year of treatment. Left: voxel-wise analysis within the first year of treatment of ET patients (year 1). Yellow-orange shows the β values (i.e., the regression coefficients) of significant regions where lower TLVC was related to faster (pseudo)atrophy. Right: the red line shows the mean voxel-wise regression between the TLVC (x-axis) and the mean edge displacement of the significant voxels from the voxel-wise analysis (y-axis). The more negative the edge displacement is, the faster the atrophy. For each decrease of 1 mL in TLVC, the edge displacement reduces by 0.031 mm (mean β of the significant voxels). ET = early treatment, TLVC = total lesion volume change.

*Voxel-wise*: For ET patients, within the first year of treatment (year 1), faster ventricular enlargement and frontal lobe atrophy were found to be associated with lower TLVC (V = 2192, mean β value of significant voxels = 0.031, Fig. 4). No significant relationship was found for DT patients in year 3. Supplementary Material Fig. 6 shows the p-value maps and the location of the peak values.

### 3.6 Research question 2c: Stable treatment period

Data points from 256 ET patients were analysed (mean age ± SD: 31.66 ± 8.46, number of male/female: 96/160). In all, 162 patients did not convert to CDMS. The same data points of the 101 DT patients as used in research question 2a were analysed.

*Whole brain:* No significant association was observed during the stable treatment period. Further, the relationship between TLVC and PBVC and between TLVC and PVVC did not differ between ET and DT patients.

**Fig. 5:**
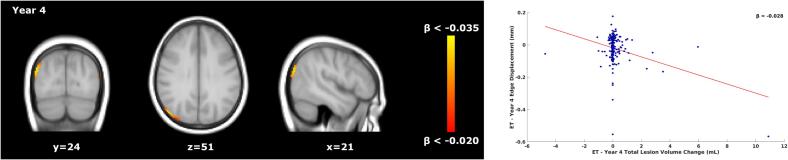
Relationship between total lesion volume change and concurrent brain atrophy in ET patients’ stable treatment period. Left: voxel-wise analysis within year 4 of the stable treatment period of ET patients. Yellow-orange shows the β values (i.e., the regression coefficients) of significant regions where higher TLVC was related to faster atrophy. Right: the red line shows the mean voxel-wise regression between the TLVC (x-axis) and the mean edge displacement of the significant voxels from the voxel-wise analysis (y-axis). The more negative the edge displacement is, the faster the atrophy. For each increase of 1 mL in TLVC, the edge displacement reduces by 0.028 mm (mean β of the significant voxels). ET = early treatment, TLVC = total lesion volume change.

*Voxel-wise*: During the stable treatment period, faster occipital lobe atrophy was associated with higher TLVC (year 4, V = 283, mean β value of significant voxels = −0.028, Fig. 5) in ET patients. For DT patients, results are summarised in Table 5 (years 4 and 5). Supplementary Material Fig. 7 shows the p-value maps and the location of the peak values.

### 3.7 Research question 3: Influence of conversion to CDMS on the relationship between atrophy and concurrent lesion activity

All available data points from the entire dataset of 387 patients were analysed.

*Whole brain:* converters and non-converters to CDMS showed significantly different relationships between PBVC and TLVC (p < 0.001). The model only revealed a significant positive relationship for patients who did not convert to CDMS (B = 0.084, SE = 0.016, p < 0.001). Similarly, the relationship between PVVC and TLVC seemed to be modulated by CDMS status (p < 0.001). The model only revealed a significant negative relationship for non-converters to CDMS (B = −0.837, SE = 0.145, p < 0.001).

**Fig. 6:**
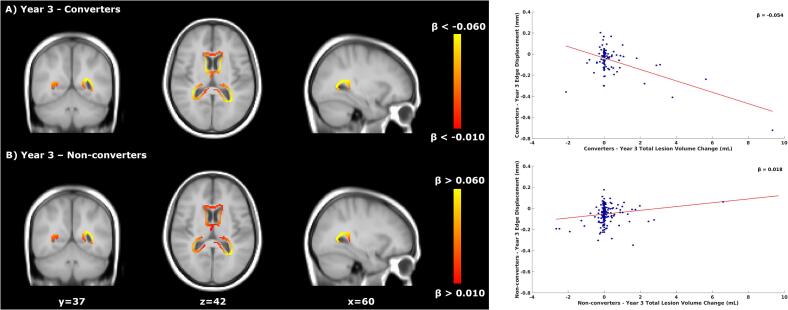
Relationship between total lesion volume change and concurrent brain atrophy in converters and non-converters to CDMS. Left: voxel-wise analysis for the difference between the converters and non-converters to CDMS (year 3). Yellow-orange shows the β values (i.e., the regression coefficients) of significant regions where higher TLVC was related to faster atrophy for converters and where lower TLVC was related to faster atrophy in non-converters. Right: the red line shows the mean voxel-wise regression between the TLVC (x-axis) and the mean edge displacement of the significant voxels from the voxel-wise analysis (y-axis). The more negative the edge displacement is, the faster the atrophy. For each increase of 1 mL in TLVC, the edge displacement reduces by 0.054 (mean β of the significant voxels) in converters to CDMS. For each decrease of 1 mL in TLVC, the edge displacement reduces by 0.018 in non-converters to CDMS. CDMS = clinically definite multiple sclerosis, TLVC = total lesion volume change.

*Voxel-wise*: Results are summarised in Table 6. In years 3 and 4, converters and non-converters to CDMS showed significantly different relationships between atrophy and TLVC (Fig. 6). In patients who did convert to CDMS, faster ventricular enlargement and occipital lobe atrophy was associated with higher TLVC. Within the same time intervals, faster atrophy was associated with lower TLVC in non-converters to CDMS. Supplementary MaterialFig. 8 shows the p-value maps and the location of the peak values.

**Table 6:** Schematic representation of the voxel-wise significant results for the influence of conversion to CDMS on the concurrent relationship between atrophy and TLVC.

Time Interval	Number of significant voxels	Location of significant voxels	MNI coordinates (X/Y/Z) of peak location	Conv mean β value (relationship)	Nonc mean β value (relationship)
Year 3	3779	PV	60/37/42	−0.054 (Faster Atrophy/Higher TLVC)	0.018 (Faster Atrophy/Lower TLVC)
Year 4	153	OL	72/25/49	−0.026 (Faster Atrophy/Higher TLVC)	0.014 (Faster Atrophy/ Lower TLVC)

Conv = converters to clinically definite multiple sclerosis (CDMS), Nonc = non-converters to CDMS, PV = periventricular, OL = occipital lobe, TLVC = total lesion volume change.

## 4 Discussion

In this post-hoc study, we found that inflammation and brain atrophy occurred simultaneously in early MS. Interestingly, the spatio-temporal correspondence between these two processes seems to take place mostly in the periventricular region. Furthermore, WM lesion activity and brain atrophy seemed to be differentially related across an untreated and treated period.

To better elucidate the complex spatio-temporal dynamics between inflammation and brain atrophy, it is important to consider two key points in the REFLEXION study design. Firstly, patients were recruited after a FCDE, implying that patients had active inflammation when entering in the study. Thus, several competitive mechanisms should be considered: the anti-inflammatory effect of the treatment, the brain atrophy (both pseudo-atrophy and “true” atrophy), and the slow focal damage accrual. Secondly, DT patients who converted to CDMS during the first two years received treatment earlier than the year 3 time point. Thus, patients who received delayed treatment from year 3 onwards were the less active cases (i.e., non-converters). Taken together, these factors may certainly influence the relationship between inflammation and atrophy.

Contrary to the studies which assumed that inflammation precedes brain atrophy (Chard et al., 2003, Paolillo et al., 2004, Dalton et al., 2002), our first relevant finding is that WM lesion activity and brain atrophy developed simultaneously over time, suggesting a possible (partly or wholly) independent development of these two processes. Few other MRI studies have investigated the relationship between WM lesion activity and brain atrophy within the same follow-up period in early MS (Dalton et al., 2004, Varosanec et al., 2015). Such studies found how WM lesion accrual and brain volume changes occurred simultaneously. Our results largely confirmed this: increased WM lesion activity was related to faster widespread atrophy within the same follow-up period. The pathological explanation for this association remains to be elucidated. Neuropathological observations revealed how profound axonal loss in the normal appearing WM seemed to develop independently from axonal injury in demyelinated lesions (DeLuca et al., 2006). Furthermore, in long-term MS patients, ongoing myelin destruction, associated with axonal and neuronal degeneration, was detected in the absence of parenchymal inflammatory infiltration (Bø et al., 2003, Peterson et al., 2001). These observations suggested that brain atrophy in MS could occur independently from inflammation (Trapp & Nave, 2008).

Inflammation and atrophy were related mostly in the periventricular area, a site with a high prevalence of MS lesions (Filippi et al., 2019, Trip and Miller, 2005). This finding is in line with other studies that showed how the presence of these two disease processes is higher in the periventricular areas and in proximity to cerebrospinal fluid (CSF) spaces (Brown et al., 2017, Liu et al., 2015). Such greater periventricular activity is related to the presence of locally secreted proinflammatory cytokines derived from CSF compartments harbouring B cells that reside within the CSF space (Magliozzi et al., 2018). Interestingly, the cerebellum and the temporal lobe showed significant correlations in the left hemisphere. To this respect, a recent work found a strong correlation between lesions and atrophy in the left hemisphere (Muthuraman et al., 2020). Further, in a recent paper analysing the first two years of our study population (Battaglini et al., 2022), a strong left-lateralization in the inflammatory activity (represented by new lesion accrual) was found. Given this context, we believe that this finding deserves further investigation to be performed on a different population.

During the untreated period of DT patients (first 2 years of the REFLEX study), whole brain analyses showed that faster global and central atrophy were related to lower TLVC. Voxel-wise analysis confirmed this result within year 1. These findings are not surprising if we consider that patients were recruited after their first attack and thus entered the study with active inflammatory processes. Thus, it can be speculated that the initial accelerated decrease in brain volume could be due to the natural resolution of oedema and shifts in fluids. In these patients and at this early stage of the trial, these changes cannot be driven by treatment as DT patients received placebo within the REFLEX study period. At voxel-wise level, having excluded the effect of active inflammation and shift in fluids of year 1, we found a different relationship between WM lesion changes and brain atrophy across the untreated and treated periods of DT patients. Faster enlargement of the ventricles (i.e., faster atrophy) was associated with higher TLVC (i.e., faster increase of lesion load) in the second year of DT patients’ untreated period. Conversely, in these same DT patients but during their “stable” treatment period (years 4 and 5), faster atrophy was related to concurrent lower TLVC (i.e., slower increase of lesion load). These findings suggested how treatment could influence the relationship between WM lesion changes and brain atrophy. A possible explanation could be that treatment initially exerts its effect on inflammation while effects on brain atrophy require more time to be detected. Whole brain analyses did not reveal any significant difference across the untreated and treated periods of DT patients, probably related to the fact that the data points from different time-intervals were pooled together in the linear mixed model analyses. Thus, an association present within a specific time-interval could to some extent be “diluted” by data points from other years. Since patients were recruited after their first attack, it can be hypothesised that the relationship observed for the full untreated period in DT patients was mainly driven by their data from year 1. To support this theory, whole brain results (i.e., faster atrophy being related to lower TLVC) were matched by voxel-wise findings in year 1, but not in year 2 of the untreated period. Given this context, the initial shift in fluid and inflammatory activity might have influenced the real relationship between WM lesion changes and brain atrophy. This in turn could have obscured the presence of any significant difference across the untreated and treated periods of the DT patients.

Another key observation in our study is that, within the first year of treatment, faster global and central brain atrophy were associated with lower TLVC. Paradoxically, anti-inflammatory drugs have often been associated with an acceleration of brain volume loss following the initiation of therapy. This phenomenon, referred to as “pseudo-atrophy”, is generally assumed to be related to resolution of inflammation and fluid shifts (De Stefano et al., 2014, Zivadinov et al., 2008). Although its dynamics are still largely unknown, pseudo-atrophy certainly complicates the interpretation of brain atrophy measurements in both clinical and research settings (De Stefano et al., 2021). Thus, it is crucial to investigate to what extent the pseudo-atrophy may be related to the resolution of inflammation as opposed to brain atrophy. Furthermore, it could be of great relevance to localise the brain tissues or regions where this phenomenon occurs. Congruent to the effects on pseudo-atrophy, the results showed that lower TLVC was associated with faster ventricular enlargement and frontal lobe atrophy within the first year of treatment for ET patients. Conversely, such relationship was not detected within the first year of treatment for DT patients (year 3). This result was confirmed by the voxel-wise statistics looking at the effect of treatment on the relationship between inflammation and brain atrophy (research question 2). Normally, one would expect that both ET and DT patients would have shown the same response to treatment onset. However, the lack of a pseudo-atrophy effect in the DT patients could be attributed to several reasons. Firstly, several studies reported that pseudo-atrophy is found mainly in patients who showed active inflammation (Radue et al., 2015, Sastre-Garriga et al., 2015, Vidal-Jordana et al., 2013). In this regard, DT patients showed relatively stable WM lesion activity (i.e., TLVC value close to 0; data not shown) during their first year of treatment. Secondly, the low sample size of the DT group should also be taken into consideration (ET: 231 patients; DT: 65 patients). Finally, and according to the REFLEXION study design, the majority of DT patients receiving treatment from year 3 were most likely the less active cases (non-converters: 59; converters: 6). Taken together, these factors might have hampered the detection of the pseudo-atrophy effect within DT patients’ first year of treatment.

To prevent the confounding effect of resolving oedema and pseudo-atrophy during the first year of treatment, we restricted our analyses to a stable treatment period, where patients had received at least one year of treatment. Significantly different relationships were found at the voxel-wise level within this stable treatment period; whereby, in ET patients, faster occipital lobe atrophy was associated with higher TLVC, whereas in DT patients faster atrophy was associated with lower TLVC as summarised in Table 5 (years 4 and 5). These results were confirmed by the voxel-wise statistics looking at treatment effect on the relationship between WM lesion activity and brain atrophy (research question 2). Although not straightforward, one might hypothesise a sort of prolonged pseudo-atrophy effect on the DT patients. Indeed, the course of pseudo-atrophy is not completely understood and thus, the assumption that pseudo-atrophy occurs only during the first year of treatment is not necessarily valid (De Stefano & Arnold, 2015).

Finally, we investigated whether conversion to CDMS could influence the relationship between inflammation and brain atrophy. Our results highlighted a different relationship across conversion groups: in non-converters faster global and central atrophy were associated with lower TLVC; in patients who did convert to CDMS, lesion activity and brain atrophy developed simultaneously, with faster ventricular enlargement and occipital lobe atrophy being related to higher TLVC. This is not surprising if we consider that increased inflammatory activity and brain atrophy are related to higher risks of conversion to CDMS (Kalincik et al., 2012, Tintoré et al., 2006). However, these results should be interpreted with caution as several factors (e.g., the switch to open-label treatment upon conversion) could have influenced the relationship between WM changes and brain atrophy.

This study is not without limitations. Firstly, in the REFLEXION study treatment and conversion status are intersected, with patients switching to open-label treatment upon conversion to CDMS. To overcome the potential confounding related to the REFLEXION study design, we restricted our analyses to specific time periods (i.e., untreated period, first year of treatment, and stable treatment). Secondly, beyond a biological reason, there could be a technical explanation why spatial correspondence between brain atrophy and lesion changes occurred mostly in the periventricular area. The atrophy voxel-wise analysis method is based on the comparison of brain parenchymal border displacements occurring at the same voxel across the studied population. The irregularity of the neocortex/CSF interface hampers a perfect alignment/overlap. Thus, the strength of the relationship between global lesion changes and voxel-wise atrophy displacement may be reduced within the cortex, due to this technical issue. The same is not true for the ventricles, usually characterised by a smoother parenchyma/CSF interface. Thirdly, we focused our analyses only on the relationships between WM lesion activity and global/central brain atrophy. Several studies have highlighted the presence of GM damage in early MS (Dalton et al., 2004, Henry et al., 2008, Raz et al., 2010). In the present work, the limited quality of the T1-weighted images and the poor contrast between tissues made it difficult to look at the relationship between WM lesion activity and GM damage. Future studies could address this issue by implementing a new generation of imaging processing methods (i.e., SIENA-XL, Jacobian integration methods) (Battaglini et al., 2018, Nakamura et al., 2013), which are able to provide robust and accurate GM volume estimates. In addition, it would be very interesting to analyse whether WM lesion accrual in specific brain tracts is related to damage in “anatomically contiguous” cortical lobes. Fourthly, multiple testing correction was not performed due to the explorative nature of the post-hoc analyses. Thus, future studies are needed to confirm the results obtained in this work. Fifthly, differences in magnetic field strength across study sites could have biased the outcomes of the analysis. However, within the REFLEXION study, all study sites were required to follow an MRI protocol to ensure standardised scanning acquisition with all the images having the same voxel dimensions and the majority of the patients analysed (i.e., 92%) being scanned using a 1.5 T scanner. Further, the eventual difference across scanners has been statistically addressed by controlling for the study site in the analyses we performed. Finally, the pathological explanation of the uncoupling between inflammation and brain atrophy remains to be elucidated. Indeed, although our results suggested that these two processes could develop independently, genetic data and observations from most experimental models appear to favour a pathogenesis model in which inflammation precedes neurodegeneration (Milo et al., 2020). Thus, the question whether inflammation and brain atrophy are causally related or could develop independently is still a topic of discussion and our results did not provide a definite solution. Future studies should focus not only on the correlations within the same time interval but should also investigate the relationships between WM lesion changes and subsequent brain atrophy and vice versa. This has been addressed in a separate paper for the present dataset (Mattiesing et al., 2022).

To conclude, we found that inflammation and brain atrophy occur simultaneously in early MS, thus suggesting how WM lesion activity contributes only partially to the loss of overall brain tissue, or vice versa. Interestingly, the periventricular regions are always affected by atrophy. The relationship between WM lesion changes and brain atrophy differed across the investigated study periods. During the first year of the study, both DT and ET patients showed the pseudo-atrophy phenomenon, corresponding to lower lesion volume changes being related to faster (pseudo)atrophy. This could probably be explained by the presence of two factors: the initial effect of treatment (in the ET group) and the natural resolution of oedema (in both the ET and DT groups) as patients showed active inflammation when entering the study. After this effect resolves over time, in the untreated period and later on during stable treatment faster “true” atrophy was associated with higher lesion volume changes.

## Data Availability Statement

Any requests for data by qualified scientific and medical researchers for legitimate research purposes will be subject to Merck’s Data Sharing Policy. All requests should be submitted in writing to Merck’s data sharing portal https://www.merckgroup.com/en/research/our-approach-to-research-and-development/healthcare/clinical-trials/commitment-responsible-data-sharing.html.

When Merck has a co-research, co-development, or co-marketing or co-promotion agreement, or when the product has been out-licensed, the responsibility for disclosure might be dependent on the agreement between parties. Under these circumstances, Merck will endeavour to gain agreement to share data in response to requests.

## Funding

The REFLEXION study was supported by Merck (CrossRef Funder ID: 10.13039/100009945).

## CRediT authorship contribution statement

**Giordano Gentile:** Conceptualization, Formal analysis, Investigation, Methodology, Visualization, Writing – original draft, Writing – review & editing. **Rozemarijn M. Mattiesing:** Conceptualization, Formal analysis, Investigation, Methodology, Visualization, Writing – original draft, Writing – review & editing. **Iman Brouwer:** Investigation, Methodology, Software, Writing – review & editing. **Ronald A. van Schijndel:** Investigation, Methodology, Software, Writing – review & editing. **Bernard M.J. Uitdehaag:** Writing – review & editing. **Jos W.R. Twisk:** Methodology, Writing – review & editing. **Ludwig Kappos:** Writing – review & editing. **Mark S. Freedman:** Writing – review & editing. **Giancarlo Comi:** Writing – review & editing. **Dominic Jack:** Writing – review & editing. **Frederik Barkhof:** Conceptualization, Funding acquisition, Investigation, Supervision, Writing – review & editing. **Nicola De Stefano:** Conceptualization, Funding acquisition, Investigation, Supervision, Writing – review & editing. **Hugo Vrenken:** Conceptualization, Funding acquisition, Investigation, Methodology, Supervision, Writing – original draft, Writing – review & editing. **Marco Battaglini:** Conceptualization, Funding acquisition, Investigation, Methodology, Supervision, Writing – original draft, Writing – review & editing.

## Declaration of Competing Interest

**RMM** has received research support from Merck. **IB** has received research support from Merck, Novartis, Teva, and the Dutch MS Research Foundation. **BMJU** reports research support and/or consultancy fees from Biogen, Merck, Novartis, Roche, Sanofi, Teva, and Immunic Therapeutics. **LK's** institution (University Hospital Basel) has received the following exclusively for research support: Steering committee, advisory board, and consultancy fees from Actelion (Janssen/J&J), Bayer, Biogen, BMS, Janssen (J&J), Merck, Novartis, Roche, Sanofi, Santhera, and TG Therapeutics; speaker fees from Bayer, Biogen, Merck, Novartis, Roche, and Sanofi; support of educational activities from Allergan, Bayer, Biogen, CSL Behring, Desitin, Merck, Novartis, Roche, Pfizer, Sanofi, Shire, and Teva; license fees for Neurostatus products; and grants from Bayer, Biogen, European Union, InnoSwiss, Merck, Novartis, Roche, Swiss MS Society, and Swiss National Research Foundation. **MSF** has received honoraria or consultation fees from Alexion, Atara Biotherapeutics, Bayer, BeiGene, BMS (Celgene), EMD Serono, Janssen (J&J), Merck, Novartis, PendoPharm, Roche, and Sanofi; has been a member of a company advisory board, board of directors, or other similar group for Alexion, Atara Biotherapeutics, Bayer, BeiGene, BMS (Celgene), Clene Nanomedicine, Janssen (J&J), McKesson, Merck, Novartis, Roche, and Sanofi; has participated in a company sponsored speaker’s bureau for EMD Serono and Sanofi; and has been in receipt of research or educational grants from Sanofi. **GC** has received consulting fees from Bayer, Biogen, Merck, Novartis, Receptos, Roche, Sanofi, and Teva; lecture fees from Bayer, Biogen, Merck, Novartis, Sanofi, Serono Symposia International Foundation, and Teva; and trial grant support from Bayer, Biogen, Merck, Novartis, Receptos, Roche, Sanofi, and Teva. **DJ** is an employee of Merck Serono Ltd, Feltham, UK (an affiliate of Merck KGaA, Darmstadt, Germany). **FB** is supported by the NIHR Biomedical Research Centre at UCLH and is a consultant to Biogen, Combinostics, IXICO, Merck, and Roche. **NDeS** is a consultant for Biogen, Merck, Novartis, Sanofi, Roche, and Teva; has grants or grants pending from FISM and Novartis, is on the speakers’ bureaus of Biogen, Merck, Novartis, Roche, Sanofi, and Teva; and has received travel funds from Merck, Novartis, Roche, Sanofi, and Teva. **HV** has received research support from Merck, Novartis, Pfizer, and Teva; consulting fees from Merck; and speaker honoraria from Novartis. All funds were paid to his institution. **GG, RAvS, JWRT,** and **MB** report no disclosures.
