# Supplementary material for: The spatio-temporal relationship between concurrent lesion and brain atrophy changes in early multiple sclerosis: A post-hoc analysis of the REFLEXION study
Source: Neuroimage Clin. 2023 Apr 5;38:103397. doi: 10.1016/j.nicl.2023.103397 (PMC10300577; doi:10.1016/j.nicl.2023.103397)
Supplement: Supplementary data 1 [file mmc1.pdf]

Supplementary Material

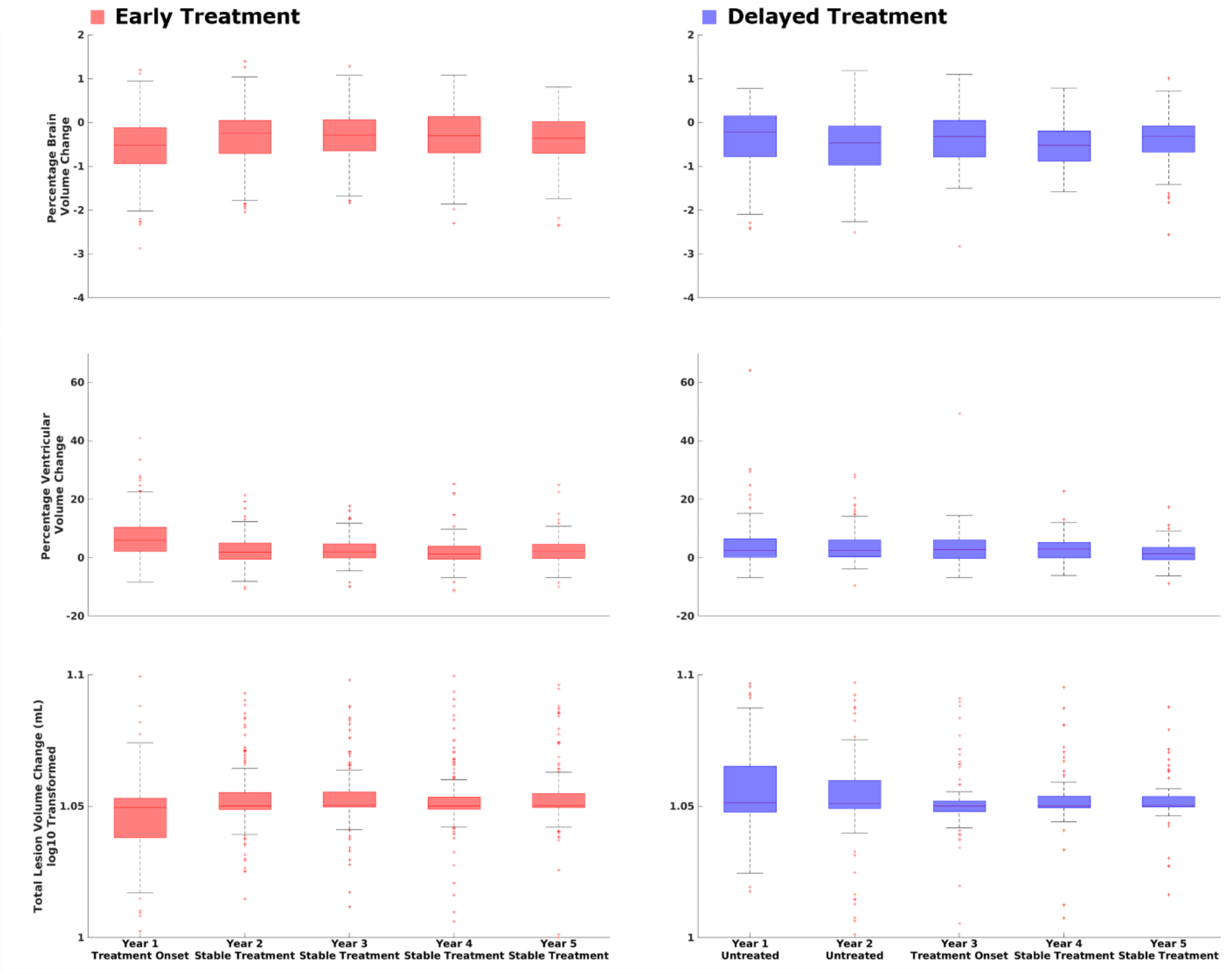

Figure 1. Percentage of brain and ventricular volume changes and total lesion volume changes across treatment groups.

Boxplots depicting the percentage brain volume change, percentage ventricular volume change, and total lesion volume change across all years for the early and delayed treatment groups. For visualization purposes, total lesion volume change values were log-transformed.

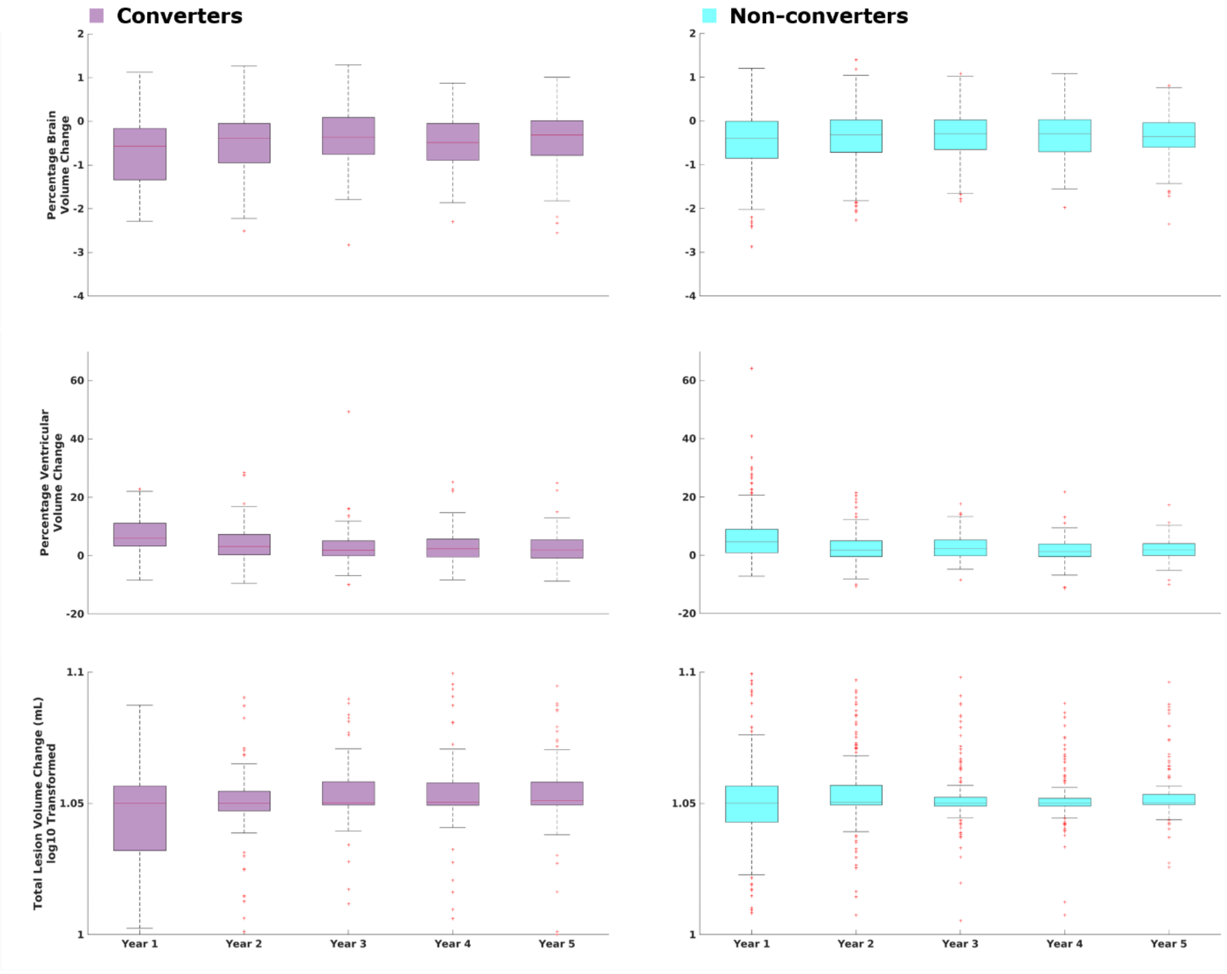

Figure 2. Percentage of brain and ventricular volume changes and total lesion volume changes across conversion groups.

Boxplots depicting the percentage brain volume change, percentage ventricular volume change, and total lesion volume change across all years for the interval-specific converters and non-converters to clinically definite multiple sclerosis. For visualization purposes, total lesion volume change values were log-transformed.

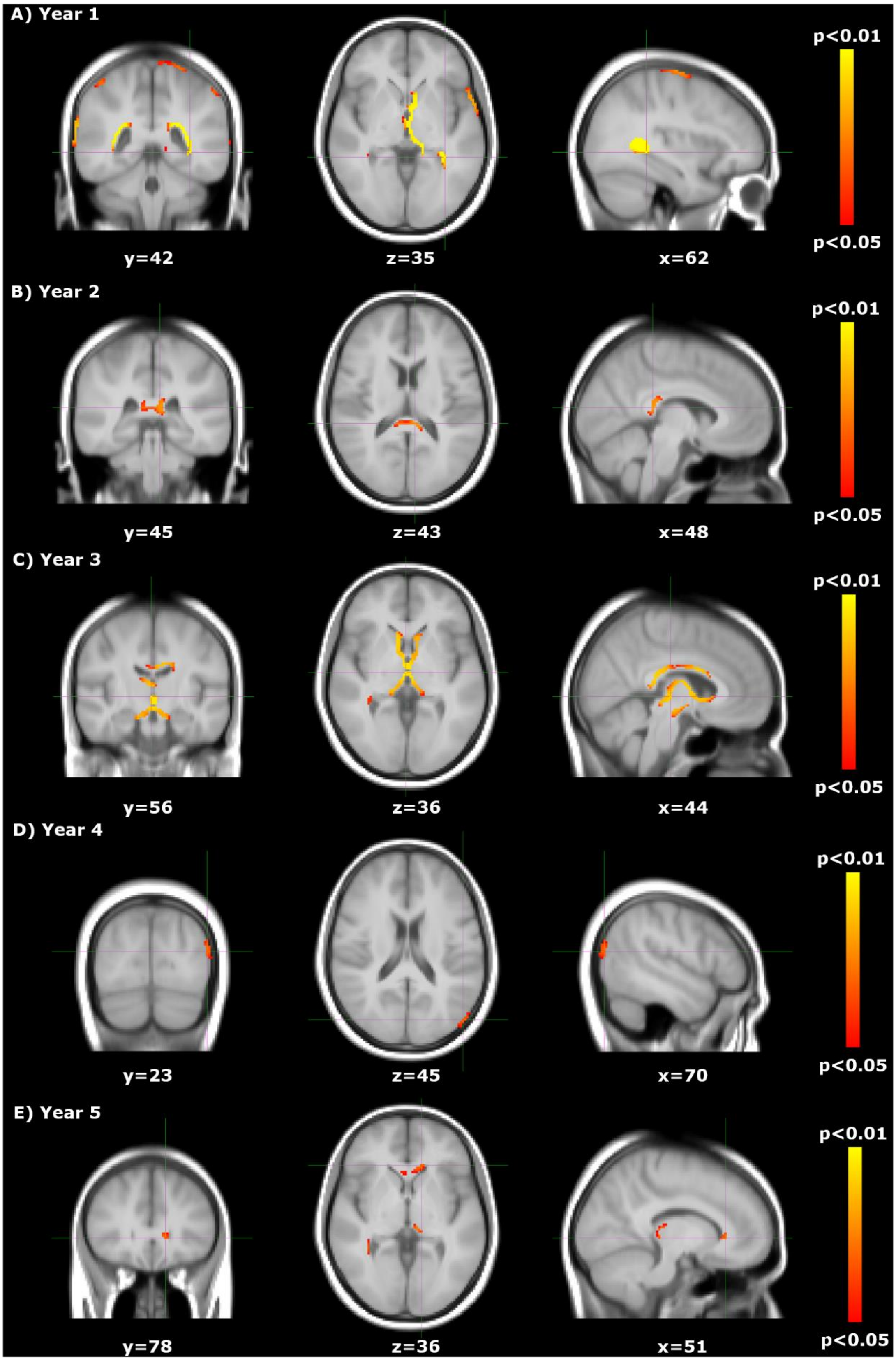

Figure 3. Location of peak values for the overall relationship between total lesion volume change and concurrent brain atrophy.

The cursor indicates the MNI coordinates of peak location of the voxel-wise analyses for the overall

relationship between total lesion volume change and concurrent brain atrophy. Yellow-orange shows voxels of significant regions where lower total lesion volume change was related to faster (pseudo)atrophy in year 1 (A) and where higher total lesion volume change was related to faster atrophy from year 2 to year 5 (B, C, D, and E).

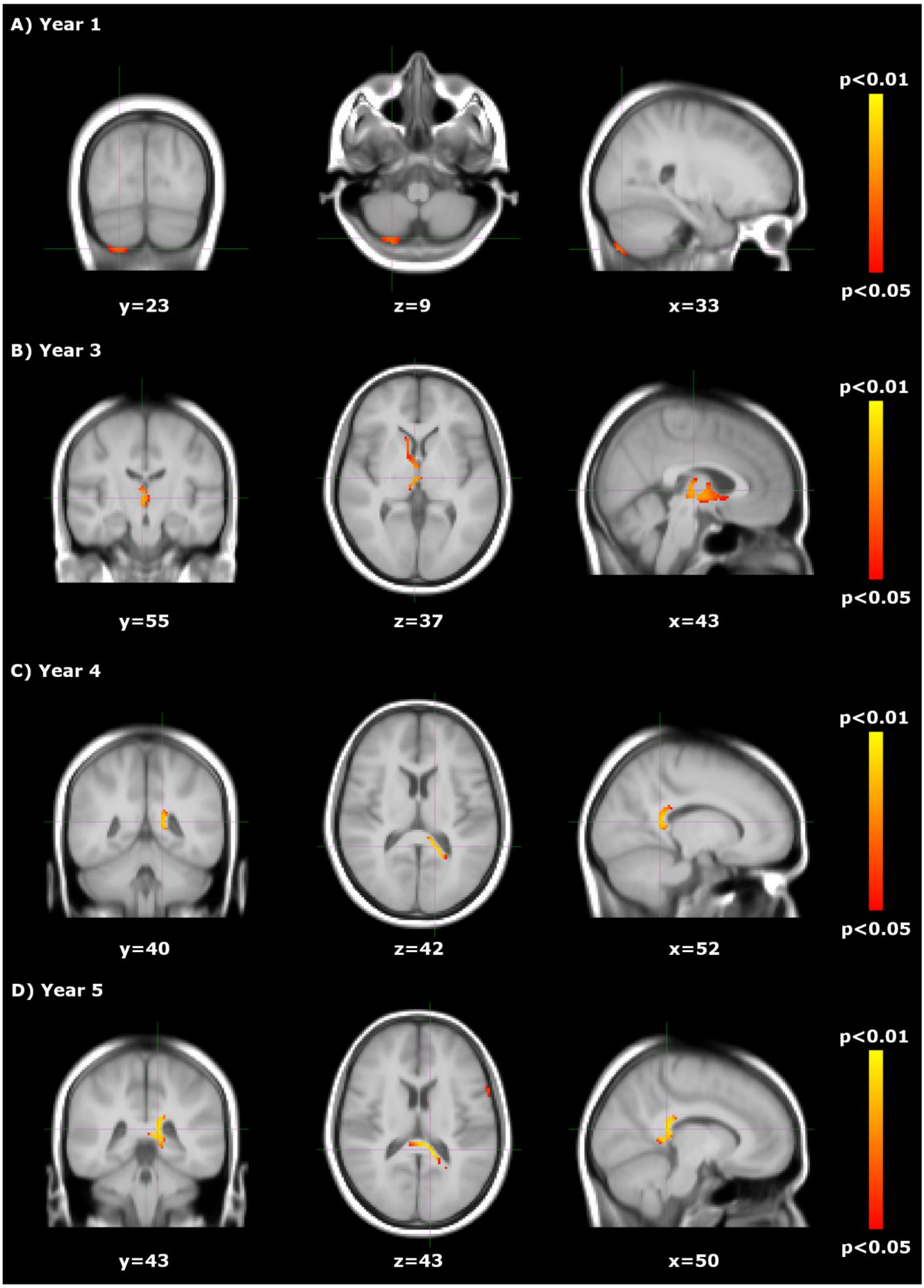

Figure 4. Location of peak values for the relationship between total lesion volume change and concurrent brain atrophy across treatment groups.

The cursor indicates the MNI coordinates of peak location of the voxel-wise analyses for the

relationship between total lesion volume change and concurrent brain atrophy across treatment groups. Yellow-orange shows voxels of significant regions where early treatment and delayed treatment patients showed significantly different relationships between atrophy and total lesion volume change.

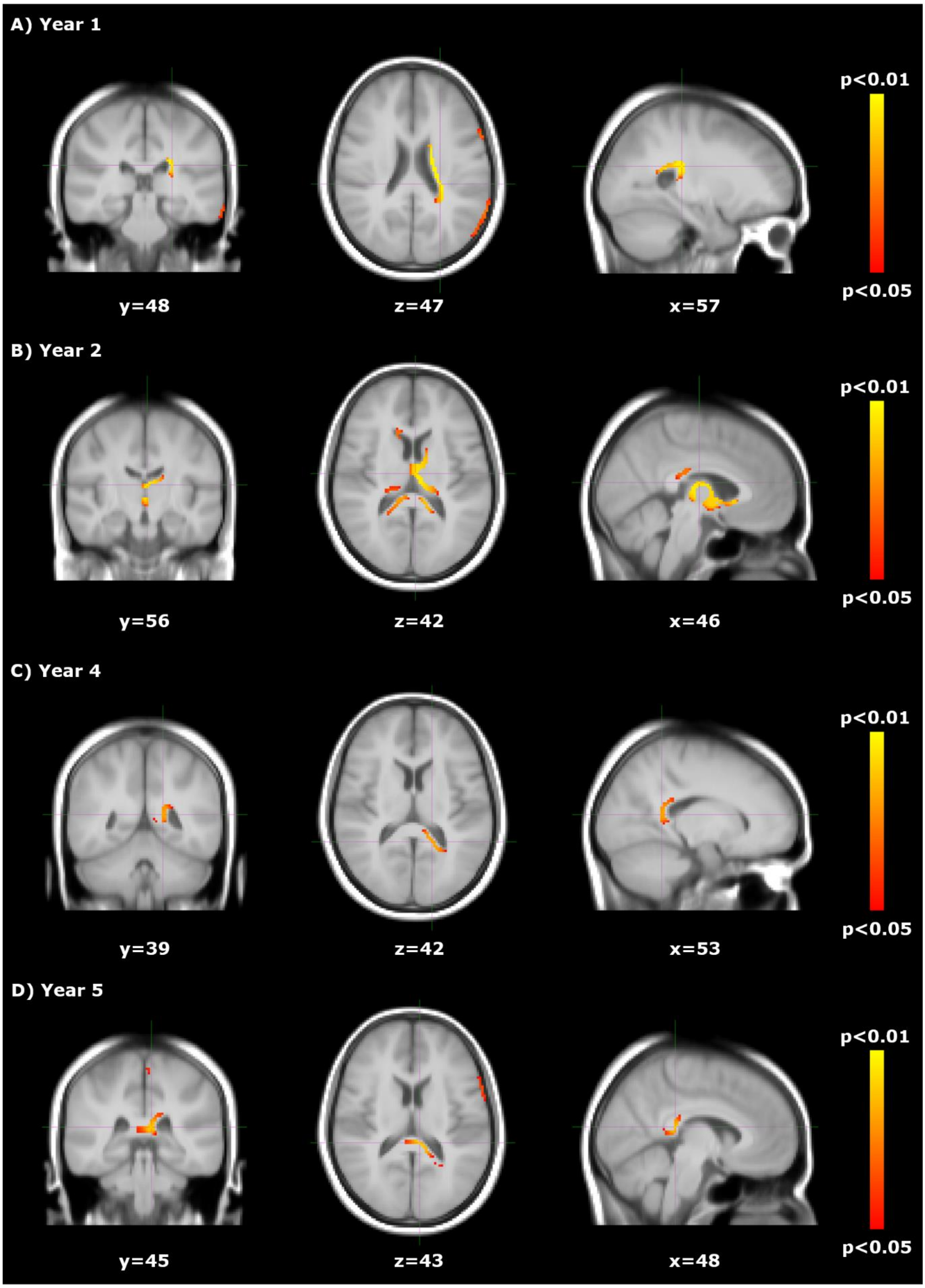

Figure 5. Location of peak values for the relationship between total lesion volume change and concurrent brain atrophy across the untreated and treated period of delayed treatment patients.

The cursor indicates the MNI coordinates of peak location of the voxel-wise analyses for the relationship between total lesion volume change and concurrent brain atrophy across the untreated (years 1 and 2) and treated period (years 4 and 5) of delayed treatment patients. Yellow-orange shows voxels of significant regions where lower total lesion volume change was related to faster atrophy in years 1, 4 and 5 (A, C, and D) and where higher total lesion volume change was related to faster atrophy in year 2 (B).

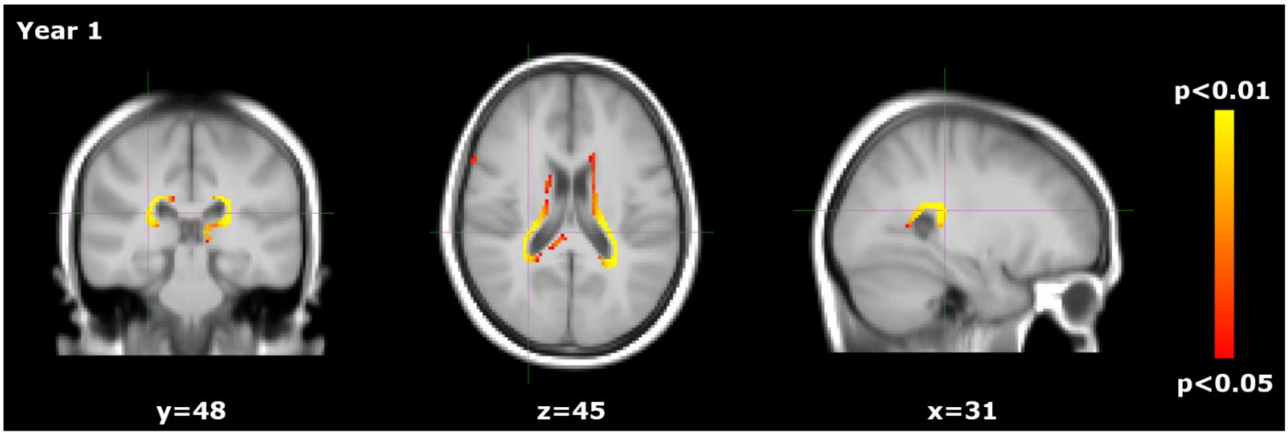

Figure 6. Location of peak values for the relationship between total lesion volume change and concurrent brain atrophy in early treatment patients' first year of treatment.

The cursor indicates the MNI coordinates of peak location of the voxel-wise analyses for the relationship between total lesion volume change and concurrent brain atrophy in early treatment patients' first year of treatment. Yellow-orange shows voxels of significant regions where lower total lesion volume change was related to faster (pseudo)atrophy.

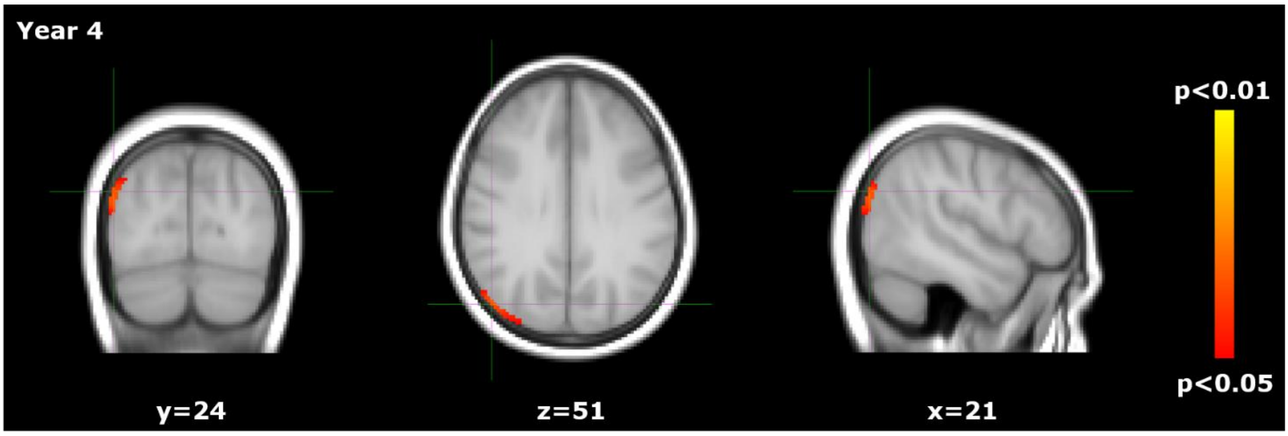

Figure 7. Location of peak values for the relationship between total lesion volume change and concurrent brain atrophy in early treatment patients' stable treatment period.

The cursor indicates the MNI coordinates of peak location of the voxel-wise analyses for the relationship between total lesion volume change and concurrent brain atrophy in early treatment patients' stable treatment period. Yellow-orange shows voxels of significant regions where higher total lesion volume change was related to faster atrophy.

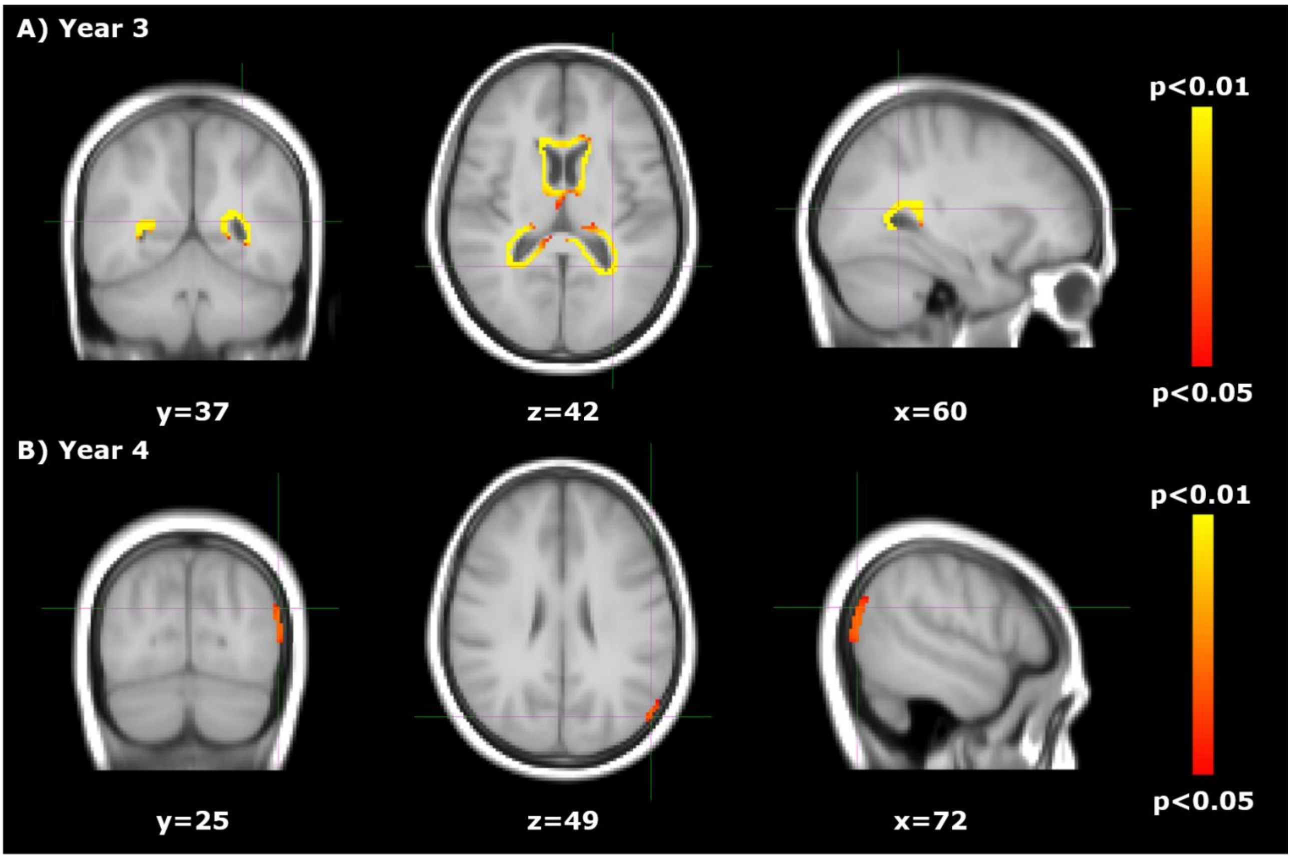

Figure 8. Location of peak values for the relationship between total lesion volume change and concurrent brain atrophy across conversion groups.

The cursor indicates the MNI coordinates of peak location of the voxel-wise analyses for the relationship between total lesion volume change and concurrent brain atrophy across converters and non-converters to clinically definite multiple sclerosis. Yellow-orange shows voxels of significant regions where converters and non-converters showed significantly different relationships between atrophy and total lesion volume change.

Table 1. Scanning protocol details.

	T1-weighted	Dual-echo (PD-/T2-weighted images)
**Sequence**	2D spin-echo	2D dual-echo spin-echo
**Plane**	Axial	Axial
**TR (ms)**	400-600	2000-3000
**TE1/TE2 (ms)**	10-16	20-30/80-100
**Number of slices**	46	46
**Slice Thickness (mm)**	3	3

mm = millimeter, ms = milliseconds, PD = proton-density, TE1/TE2 = echo time 1/2, TR = repetition time.

Table 2. Equations of the linear mixed statistical models.

<table>
<tr><th>Research Question</th><th>Period</th><th>Equation</th></tr>
<tr><td>1</td><td>Whole study</td><td>$PBVC_{ijk}$ or $PVVC_{ijk} = \beta1_j TLVC_{ijk} + \beta2_j Age_{jk} + \beta3_j Sex_{jk} + \beta4_j \text{Interval-specific CDMS status}_{ijk} + \beta5_j Treatment_{jk} + B0_{jk}$</td></tr>
<tr><td>2</td><td>Whole study</td><td rowspan="3">$PBVC_{ijk}$ or $PVVC_{ijk} = \beta1_j TLVC_{ijk} + \beta2_j Age_{jk} + \beta3_j Sex_{jk} + \beta4_j \text{Interval-specific CDMS status}_{ijk} + \beta5_j Treatment_{jk} + \beta6_j TLVC_{ijk} * Treatment_{jk} + B0_{jk}$</td></tr>
<tr><td>2b</td><td>First year of treatment</td></tr>
<tr><td>2c</td><td>Stable treatment</td></tr>
<tr><td rowspan="3">2a</td><td>DT untreated</td><td>$PBVC_{ijk}$ or $PVVC_{ijk} = \beta1_j TLVC_{ijk} + \beta2_j Age_{jk} + \beta3_j Sex_{jk} + B0_{jk}$</td></tr>
<tr><td>DT treated</td><td>$PBVC_{ijk}$ or $PVVC_{ijk} = \beta1_j TLVC_{ijk} + \beta2_j Age_{jk} + \beta3_j Sex_{jk} + \beta4_j \text{Interval-specific CDMS status}_{ijk} + B0_{jk}$</td></tr>
<tr><td>DT untreated versus treated</td><td>$PBVC_{ijk}$ or $PVVC_{ijk} = \beta1_j TLVC_{ijk} + \beta2_j Age_{jk} + \beta3_j Sex_{jk} + \beta4_j \text{Interval-specific CDMS status}_{ijk} + \beta5_j Period_{ijk} + \beta6_j TLVC_{ijk} * Period_{ijk} + B0_{jk}$</td></tr>
<tr><td>3</td><td>Whole study</td><td>$PBVC_{ijk}$ or $PVVC_{ijk} = \beta1_j TLVC_{ijk} + \beta2_j Age_{jk} + \beta3_j Sex_{jk} + \beta4_j \text{Interval-specific CDMS status}_{ijk} + \beta5_j Treatment_{jk} + \beta6_j TLVC_{ijk} * \text{Interval-specific CDMS status}_{ijk} + B0_{jk}$</td></tr>
</table>

In each statistical model, a three-level structure was used to account for the dependency of the yearly repeated measurements (i). The $B0_{jk}$ is the random intercept that reflects that observations were clustered within patients (j) and patients within study sites (k). β = slope coefficient, CDMS = clinically definite multiple sclerosis, DT = delayed treatment, PBVC = percentage brain volume change, PVVC = percentage ventricular volume change, TLVC = total lesion volume change.
